# Tailored vs. Standardized Internet-Based Cognitive Behavior Therapy for Depression and Comorbid Symptoms: A Randomized Controlled Trial

**DOI:** 10.1371/journal.pone.0036905

**Published:** 2012-05-15

**Authors:** Robert Johansson, Elin Sjöberg, Magnus Sjögren, Erik Johnsson, Per Carlbring, Therese Andersson, Andréas Rousseau, Gerhard Andersson

**Affiliations:** 1 Department of Behavioural Sciences and Learning, Linköping University, Linköping, Sweden; 2 Department of Psychology, Umeå University, Umeå, Sweden; 3 Psychiatric Clinic, University Hospital of Linköping, Linköping, Sweden; 4 Swedish Institute for Disability Research, Linköping University, Linköping, Sweden; 5 Department of Clinical Neuroscience, Psychiatry Section, Karolinska Institutet, Stockholm, Sweden; University of Missouri-Kansas City, United States of America

## Abstract

**Background and Aims:**

Major depression can be treated by means of cognitive behavior therapy, delivered via the Internet as guided self-help. Individually tailored guided self-help treatments have shown promising results in the treatment of anxiety disorders. This randomized controlled trial tested the efficacy of an Internet-based individually tailored guided self-help treatment which specifically targeted depression with comorbid symptoms. The treatment was compared both to standardized (non-tailored) Internet-based treatment and to an active control group in the form of a monitored online discussion group. Both guided self-help treatments were based on cognitive behavior therapy and lasted for 10 weeks. The discussion group consisted of weekly discussion themes related to depression and the treatment of depression.

**Methods:**

A total of 121 participants with diagnosed major depressive disorder and with a range of comorbid symptoms were randomized to three groups. The tailored treatment consisted of a prescribed set of modules targeting depression as well as comorbid problems. The standardized treatment was a previously tested guided self-help program for depression.

**Results:**

From pre-treatment to post-treatment, both treatment groups improved on measures of depression, anxiety and quality of life. The results were maintained at a 6-month follow-up. Subgroup analyses showed that the tailored treatment was more effective than the standardized treatment among participants with higher levels of depression at baseline and more comorbidity, both in terms of reduction of depressive symptoms and on recovery rates. In the subgroup with lower baseline scores of depression, few differences were seen between treatments and the discussion group.

**Conclusions:**

This study shows that tailored Internet-based treatment for depression is effective and that addressing comorbidity by tailoring may be one way of making guided self-help treatments more effective than standardized approaches in the treatment of more severe depression.

**Trial Registration:**

Clinicaltrials.gov NCT01181583

## Introduction

Major depressive disorder is now considered a world-wide problem, especially in middle- and high-income countries [1]. Several different psychological treatments exist, which are considered to be fairly equivalent in terms of efficacy [2]. Computerized cognitive behavior therapy and Internet-delivered psychological treatments are available for several psychiatric disorders [3]. Recent meta-analyses have found small to moderate effects of computerized treatments for depression and anxiety disorders [4], [5].

Research clearly suggests that comorbidity is the rule rather than the exception when it comes to major depression. For example, it has been found that comorbid anxiety syndromes such as social phobia occur frequently, and it has been found that at least 50% of depressed persons also fulfill the diagnostic criteria of an anxiety disorder [6].

Related to comorbidity with depression is depression severity. It is known that increased levels of depression are associated with higher prevalence of comorbidity of e.g. anxiety disorders and substance abuse [7]. Depression severity is also known to be a significant factor in the treatment of depression. For example, there are some evidence that there is a difference in efficacy between two forms of cognitive behavioral therapy in the treatment of the more severely depressed patients [8]. Another result related to depression severity is that there are indications that the difference between antidepressant medication and placebo is evident in severe depression, but not in mild to moderate depression [9]. These results suggest that baseline depression severity may moderate response even in different variants of Internet-delivered CBT (ICBT).

Tailoring the treatment to the clients' need could be one way to address comorbidity. The procedure of tailoring is encouraged in various ways in face-to-face CBT [10], [11], but is less common in ICBT. While tailoring has been used in ICBT to some degree, e.g. for tinnitus [12], depression [13], panic [14] and anxiety disorders [15], it has to our knowledge never been directly compared to standardized (non-tailored) ICBT in a randomized controlled trial. Tailoring in ICBT typically combines modules from different treatment packages, resulting in different prescriptions for different patients, depending on primary diagnosis and comorbidity (e.g. [15]).

Another way of treating disorders with comorbidity is to use unified treatments, where all patients are provided with the same protocol but the protocol itself is constructed to fit a broader range of patients [16]. Recently, there have been evidence of the efficacy of unified ICBT treatments for depression and anxiety disorders [17], [18].

The aim of this trial was to investigate the effects of an individually tailored ICBT treatment which directly targeted both depression and comorbid symptoms. The treatment was based on treatment modules from previous treatment protocols and were individually prescribed to the participants. We compared the tailored treatment both to standardized treatment and to an active control group in the form of a monitored online discussion group, which focused on depression. An effect was expected for both CBT treatments, where a larger effect was expected for the tailored treatment. While it has been found that online support groups can have a small effect [19], we expected that this effect would be smaller than in the treatment conditions. We also included a 6-month follow-up after completion of the treatment.

## Methods

### Ethics statement

The study was approved by the Regional Ethics Board of Linköping, Sweden. Written informed consent was obtained from all participants by surface mail.

### Recruitment and selection

The participants were recruited from an online waiting list for people interested in Internet-based treatment for depression. There was also an advertisement made in a large Swedish newspaper the week before the study formally started. Those who were interested were directed to a web page with information about the study, the treatments being tested and the therapists. From there it was possible to make an application for participation in the study.

The selection process started with an online screening. All participants answered online versions of the Beck Depression Inventory-II (BDI–II; [20]), the self-rated version of the Montgomery-Åsberg Depression Rating Scale (MADRS-S; [21]), the Beck Anxiety Inventory (BAI; [22]) and the Quality of Life Inventory (QOLI; [23]). The outcome measures used have established good psychometric properties, also when administered via the Internet [24], [25]. The results from the online screening procedure were later used as pre-treatment assessment for those included in the study.

To cover comorbidity, a set of diagnostic screening questions were given. The questions were given online in self-report format according to a decision tree structure inspired by the PrimeMD [26]. Areas covered were depression, panic disorder, generalized anxiety disorder, social phobia, stress and insomnia. There were also some additional demographic questions and questions on alcohol consumption.

An algorithm in the screening system marked a participant as potentially having a diagnosis and/or a specific problem when answering according to a predefined criteria. To be marked with potential depression, panic disorder, generalized anxiety disorder or social phobia the participant had to answer affirmative to a set of question similar to the diagnostic criteria from the DSM-IV. The screening for stress and insomnia worked slightly different. If confirming these problems by answering affirmative to a set of screening questions, the participants were directed to online version of the Perceived Stress Scale (PSS; [27]) and the Insomnia Severity Index (ISI; [28]). To be marked as having problems with stress or insomnia a participant had to answer above a pre-set score on these measures. The cutoffs used were 15 for the ISI [28] and 25 for the PSS [29].

Inclusion criteria for the study were a) being at least 18 years old, b) having a total of >14 on MADRS-S and c) a total of <36 on MADRS-S, d) <5 on MADRS-S item 9 (about suicidal ideations), e) reported unchanged dosage of medication for depression and anxiety during the last three months, f) reported no concurrent psychological treatment, g) not suffering from a severe psychiatric condition that could interfere with the treatment (e.g. bipolar disorder or schizophrenia, measured in a clinical interview), h) not having other primary medical problems which would need other treatments first hand, i) not having severe alcohol problems, j) a diagnosis of major depressive disorder according to the DSM-IV, with a current acute episode of depression or an episode in partial remission.

The diagnosis of major depressive disorder was confirmed using the Structured Clinical Interview for DSM-IV–Axis I disorders (SCID-I; [30]). The interviews were conducted by telephone by seven MSc clinical psychology students and one medical student. All interviewers were trained in the diagnostic procedures using SCID-I. While the interviewers were not blind to the results from the online screening, the diagnostic interviews were similar for all participants.

The senior researcher (licensed psychotherapist) reviewed all the protocols from the interviews together with a psychiatrist and the interviewers. Issues of medication and psychiatric history that came up in the interview were considered before inclusion was made.

**Figure 1:**
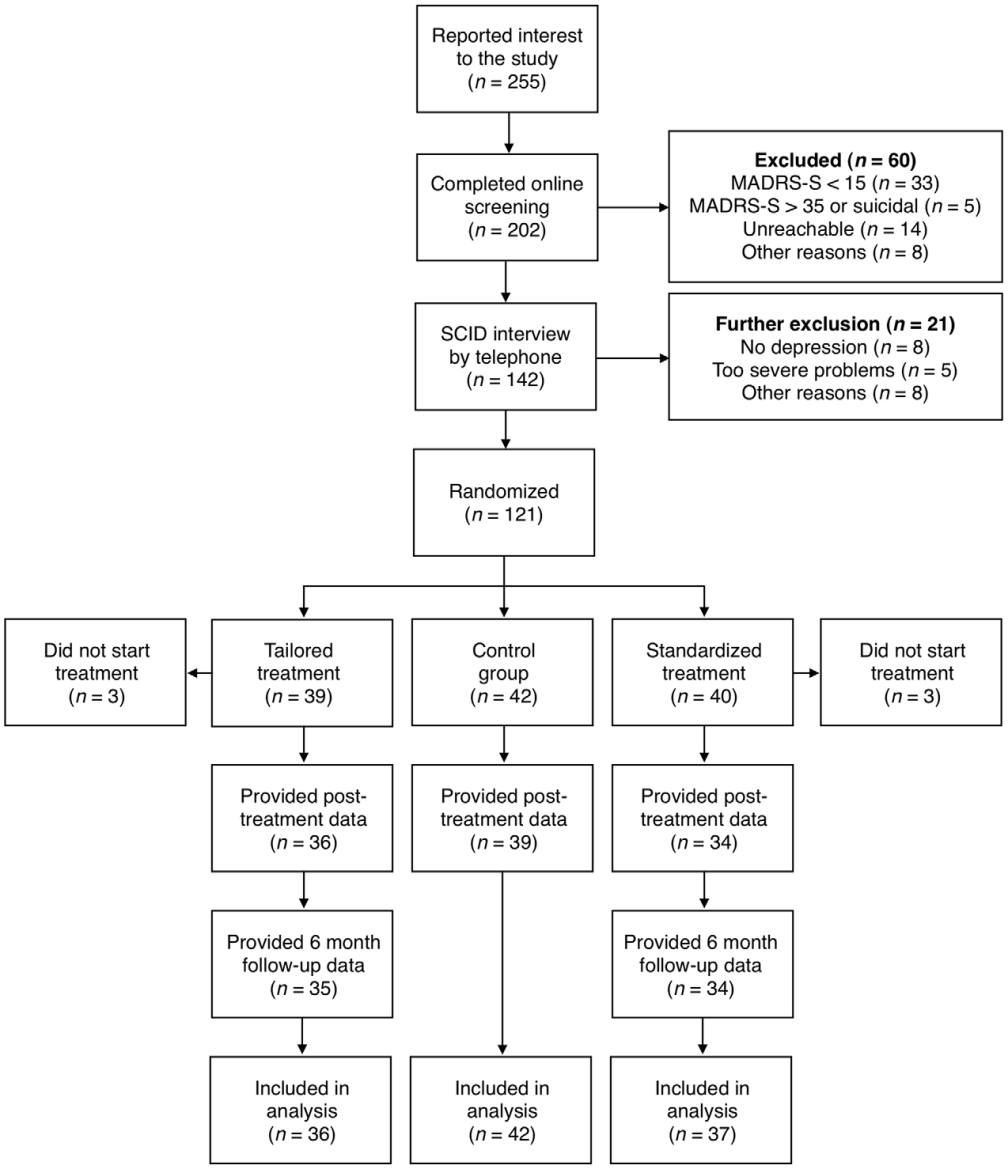
Participant flow and reasons for dropping out throughout the trial. Abbreviations: MADRS-S: Montgomery-Åsberg Depression Rating Scale–Self-rated version; SCID: Structured Clinical Interview for DSM-IV Axis I Disorders.

Of the 255 individuals who initially expressed interest in the study, 121 were subsequently included after the SCID-I had been conducted. The reasons for exclusion are specified in the flowchart found in Figure 1.

Among the randomized participants there were 71.1% women (*n* = 86) and 28.9% men (*n* = 35). The mean age was 45 years (*SD* = 12.1) ranging from 20 to 75 years. Sixty-two percent (*n* = 75) had an experience of previous psychological treatment or were in a treatment which was not considered to interfere with the study (e.g. supportive care). Sixty-nine percent (n = 84) were on medication or had a history of taking medication. See Table 1 for additional demographical data. There were no significant between-group differences in demographics. Regarding the outcome measures, there were no significant differences on any of the measures of depression and anxiety at baseline (all *F's*<0.506, all *p's*>.60). There was a tendency to pre-treatment differences on the QOLI, but not significant (*F*(2,118)  = 2.60, *n* = .079).

**Table 1:** Demographic description of the participants at randomization.

		Tailored	Standardized	Control	Total
Gender	Female	29 (74.4%)	28^a^ (70%)	29 (69%)	86 (71.1%)
	Male	10 (25.6%)	12 (30%)	13^a^ (31%)	35 (28.9%)
Age	Mean (SD)	45.7 (10.9)	43.7 (13.7)	44.8 (11.8)	44.7 (12.1)
	Min-Max	22–68	20–70	21–75	20–75
Marital status	Married	16 (41%)	18 (45%)	25 (59.5%)	59 (48.8%)
	Single	21 (53.8%)	14 (35%)	14 (33.3%)	49 (40.5%)
	Other	2 (5.1%)	8 (20%)	3 (7.1%)	13 (10.7%)
Highest educational level	Nine year compulsory school	2 (5.1%)	0	1 (2.4%)	3 (2.5%)
	Secondary school (compl.)	6 (15.4%)	14 (35%)	15 (35.7%)	35 (28.9%)
	College/university (not compl.)	7 (17.9%)	9 (22.5%)	3 (7.1%)	19 (15.7%)
	College/university (compl.)	23 (59%)	17 (42.5%)	23 (54.8%)	63 (52.1%)
	Other	1 (2.6%)	0	0	1 (0.8%)
Employment status	Employed	25 (64.1%)	24 (60%)	31 (73.8%)	80 (66.1%)
	Unemployed	4 (10.3%)	4 (10%)	3 (7.1%)	11 (9.1%)
	Student	2 (5.1%)	6 (15%)	2 (4.8%)	10 (8.3%)
	Retired	5 (12.8%)	5 (12.5%)	2 (4.8%)	12 (9.9%)
	Other	3 (7.7%)	0	3 (7.1%)	6 (5.0%)
Medication	None	15 (38.5%)	10 (25%)	12 (28.6%)	37 (30.6%)
	Earlier	15 (38.5%)	18 (45%)	13 (31%)	46 (38%)
	Present	9 (23.1%)	12 (30%)	17 (40.5%)	38 (31.4%)
Psychological treatment	None	18 (46.2%)	11 (27.5%)	17 (40.5%)	46 (38%)
	Earlier	19 (48.7%)	26 (65%)	22 (52.4%)	67 (55.4%)
	Present	2 (5.1%)	3 (7.5%)	3 (7.1%)	8 (6.6%)
Depression	In acute episode	31 (79.5%)	27 (67.5%)	31 (73.8%)	89 (73.6%)
	In partial remission	8 (20.5%)	13 (32.5%)	11 (26.2%)	32 (26.4%)
	Dysthymia	6 (15.4%)	4 (10.0%)	7 (16.7%)	17 (14.0%)
Comorbidity	Panic	6 (15.4%)	9 (22.5%)	5 (11.9%)	20 (16.5%)
	Worry	11 (28.2%)	7 (17.5%)	10 (23.8%)	28 (23.1%)
	Social fear	14 (35.9%)	13 (32.5%)	9 (21.4%)	36 (29.8%)
	Any anxiety	24 (61.5%)	22 (55.5%)	20 (47.6%)	66 (54.5%)
	Stress	32 (82.1%)	28 (70.0%)	32 (76.2%)	92 (76.0%)
	Sleep	15 (38.5%)	15 (37.5%)	18 (42.9%)	48 (39.7%)
	Any	38 (97.4%)	35 (87.5%)	38 (90.5%)	111 (91.7%)

a Two participants (one from the standardized treatment group and one from control), described themselves as “transgender” but are reported here according to their biological gender.

### Comorbidity

The screening procedure described above provided a measure of comorbidity. As seen in Table 1, stress and insomnia were most prevalent (76.0% and 39.7% respectively). A smaller proportion (29.8%) presented problems of social anxiety, 23.1% had problems with worry, and 16.5% had symptoms of panic disorder. More than half of the sample (54.5%) had a potential comorbid anxiety disorder and a large majority of the participants (91.7%) had any comorbid problem. The mean number of comorbid problems was *M* = 1.85 (*SD* = 1.04).

### Treatments and therapists

Both CBT treatments were given as guided self-help which meant that the participants downloaded self-help chapters which they worked on by themselves with e-mail support from a therapist. The delivery of the text chapters and the e-mail contact with the therapist were carried out in an online environment, which was secured both by a password and one-time codes which was sent to the participants by surface mail. All chapters had text information and exercises. For example, the material on cognitive restructuring given in both treatments contained information about the cognitive model of depression and exercises on how to register and challenge negative automatic thoughts. The participants had continuous contact with a therapist by e-mail. Most of the contact was related to feedback on exercises, but the participants were encouraged to contact the therapist in other issues (e.g. when not understanding the text material) and was guaranteed an answer within 24 hours during workdays. The therapists also sent e-mails to the participants if there had not been any contact for a week. This version of guided self-help has been described further elsewhere, e.g. in [3].

The standardizeed treatment consisted of eight self-help chapters, containing behavioral activation, cognitive restructuring, sleep management, general health advice and relapse prevention. The material has previously been tested in two randomized controlled trials [31], [32]. Even though the treatment contained eight chapters, it lasted for 10 weeks, which meant that the participant stayed in contact with the therapist for all 10 weeks and could work with some chapters longer than a week and still finish in time.

The tailored treatment consisted of a total of 25 chapters with treatment material on depression, panic, social anxiety, worrying and additional material e.g. on stress management, concentration problems, problem solving strategies, mindfulness and relaxation. All chapters were based on CBT principles. An individualized treatment plan was prepared for each participant randomized to the tailored treatment. The treatment plans were formed by discussion in the research group and were mainly based on the SCID interview and results from self-report measures. All treatment plans were made to last for 10 weeks. The average number of treatment chapters assigned to the participants was 9.7 (*SD* = 0.65), ranging from 8 to 10.

The therapists were seven MSc-level clinical psychologist students who had received clinical training. During the whole study the therapists had continuous supervision from an experienced psychotherapist. The participants were randomized to the therapists. The number of participants that each therapist was responsible for ranged from 9 to 13.

### Active control group

The participants who were randomized to the active control group were invited to participate in a moderated online discussion group during the waiting period of 10 weeks. Every week a new discussion topic was presented by the moderator. The topics were all in some way related to depression and/or treatment of depression. The participants were encouraged to use the discussion group during the treatment period. A few weeks after the treatment groups had finished their treatment, the control group received the standardized treatment, with support given when needed. At follow-up, all participants had received or had been offered treatment. However, the present analysis only contains data from the control group up the point before taking part of the standardized treatment.

### Procedure and design

The protocol for this trial and supporting CONSORT checklist are available as supporting information; see Checklist S1 and Protocol S1. Participants were allocated using an online randomization tool (www.random.org), handled by an independent person who was separate from the staff conducting the study. All measures that were collected before the treatment started were also included at mid-treatment, post-treatment and at 6 months follow-up. At post-treatment and at follow-up a structured telephone interview was also conducted. The purpose of the interview was to give an estimation of global improvement, measured by the 7-point version of the Clinical Global Impression–Improvement scale (CGI-I; [33]). The interviewers had no association to the research project and were blind to which group the participants had been randomized.

### Subgroups based on baseline depression severity

All randomized participants were classified into either higher or lower severity of depression. These classes were formed based on median baseline scores on the BDI–II. Participants with an initial depression score of BDI–II >24 (*n* = 60) were classified as higher severity and those with BDI–II <25 (*n* = 61) as lower severity. There were significant baseline differences on all outcome measures between the two classes (all *t's*>4.68; all *p's*<.001). The mean number of comorbid problems was also higher in the high severity group (*M* = 2.32 compared to *M* = 1.39; *t*(119)  = 5.44, *p*<.001). Comorbid anxiety disorders were more prevalent in the high severity group (all χ2's>6.19; all *p's*<0.05). Insomnia and stress problems had a tendency to be more prevalent among participants with higher initial severity, but did not reach statistical significance (*p* = .053 and *p* = .15 respectively).

### Data analysis

Group differences in demographic data, pre-treatment measures and in clinical significant improvement were tested using chi-square tests and one-way analysis of variance. Data from the three groups were collected before treatment, five weeks into the treatment, after treatment and at 6-month follow-up. The data was analyzed using mixed effects models, given their ability to handle missing data [34]. Analyses comparing all three groups used the pre-, mid- and post-treatment data while analyses comparing the two ICBT treatments also included the follow-up data. Between-group differences at post-treatment and at follow-up were analyzed using independent t-tests. Analyses concerning the subgroups with higher and lower pre-treatment depression severity were done separately. All analyses were performed in SPSS version 19.0 (SPSS, Inc., Chicago, IL). Six participants were excluded from the analyses since they did not start treatment. In addition, one participant from the tailored treatment group scored close to maximum on all measures at follow-up, resulting in a total score more than three standard deviations from the rest of the participants. The participant was therefore marked as an outlier and the follow-up scores were marked as missing.

Recovery after treatment was investigated using the BDI–II. On the BDI–II, recovery was defined as a post-treatment score ≤10. This definition is in line with previous clinical trials on depression (e.g. [8], [35]). The participants who did not provide post-treatment data were classified as non-recoverers. Within- and between-group effect sizes were calculated by dividing the differences in means by the pooled standard deviations [36].

## Results

Results from the mixed-effects model analyses are presented below. In all analyses, random intercept models were used and a Maximum Likelihood method and a covariance type based on the variance components were employed to provide the estimates. Means, standard deviations and effect sizes within and between groups for all self-report measures are presented in Table 2. The between-group effect sizes (Cohen's *d*) can be interpreted as follows: an effect size in the range of 0.20–0.49 is small, while 0.50–0.79 is moderate, and an effect size over 0.80 is large [37].

**Table 2:** Means, SDs and effect sizes (Cohen's d) for measures of depression, anxiety and quality of life.

		Pre		Post		Effect sizes Post			Follow-up		Effect sizes FU	
Measure	Group	n	Mean (SD)	n	Mean (SD)	Pre to Post	TA vs ST	TA/ST vs CO	n	Mean (SD)	Pre to FU	TA vs ST
Total												
BDI–II	TA	36	26.44 (7.6)	36	13.78 (9.4)	1.48	0.23	0.84	35	13.00 (9.7)	1.55	0.27
	ST	37	25.30 (8.0)	34	16.06 (10.4)	0.98		0.57	34	15.71 (10.4)	1.01	
	CO	42	26.24 (7.9)	39	21.67 (9.5)	0.51						
MADRS-S	TA	36	22.86 (3.9)	36	13.81 (6.8)	1.54	0.19	0.80	35	12.80 (7.6)	1.57	0.21
	ST	37	22.46 (5.7)	34	15.21 (7.7)	1.06		0.58	34	14.44 (8.3)	1.08	
	CO	42	23.40 (4.7)	39	19.67 (7.8)	0.55						
BAI	TA	36	14.08 (5.7)	36	9.69 (5.8)	0.76	0.39	0.69	35	8.74 (6.3)	0.90	0.36
	ST	37	16.59 (9.2)	34	12.41 (7.9)	0.53		0.28	34	11.38 (8.3)	0.63	
	CO	42	15.74 (8.1)	39	14.69 (8.4)	0.05						
QOLI	TA	36	−0.77 (1.3)	36	0.69 (1.8)	0.90	0.05	0.25	35	0.82 (1.9)	0.89	0.05
	ST	37	−0.28 (1.7)	34	0.79 (1.8)	0.59		0.30	34	0.72 (1.9)	0.54	
	CO	42	−0.15 (1.5)	39	0.24 (1.8)	0.22						
High level of depression												
BDI–II	TA	18	32.78 (4.6)	18	14.11 (10.7)	2.19	0.51	1.29	18	15.56 (11.2)	1.76	0.69
	ST	17	32.12 (6.6)	16	19.25 (9.4)	1.57		0.82	16	22.69 (9.2)	1.21	
	CO	23	31.74 (6.2)	21	26.81 (9.0)	0.65						
MADRS-S	TA	18	24.33 (3.6)	18	13.17 (5.7)	2.21	0.56	1.54	18	14.00 (8.2)	1.37	0.82
	ST	17	26.29 (4.9)	16	16.94 (7.8)	1.51		0.83	16	20.31 (7.1)	1.12	
	CO	23	25.70 (4.7)	21	23.00 (6.9)	0.43						
BAI	TA	18	17.67 (5.2)	18	10.33 (5.5)	1.37	0.47	1.47	18	9.11 (5.3)	1.63	0.82
	ST	17	20.06 (9.7)	16	13.56 (8.1)	0.70		0.81	16	14.62 (8.1)	0.58	
	CO	23	19.70 (7.5)	21	19.52 (6.8)	−0.07						
QOLI	TA	18	−1.07 (1.2)	18	0.97 (1.9)	1.24	0.37	1.02	18	0.66 (2.0)	1.00	0.45
	ST	17	−1.10 (1.4)	16	0.28 (1.9)	0.84		0.63	16	0.18 (1.7)	0.61	
	CO	23	−0.97 (1.3)	21	−0.77 (1.5)	0.14						
Low level of depression												
BDI–II	TA	18	20.11 (3.6)	18	13.44 (8.2)	1.01	−0.02	0.31	17	10.29 (7.1)	1.62	−0.11
	ST	20	19.50 (2.9)	18	13.22 (10.7)	0.64		0.28	18	9.50 (7.1)	1.73	
	CO	19	19.58 (3.2)	18	15.67 (5.9)	0.78						
MADRS-S	TA	18	21.39 (3.6)	18	14.44 (7.9)	0.98	−0.10	0.18	17	11.53 (7.0)	1.74	−0.38
	ST	20	19.20 (4.1)	18	13.67 (7.5)	0.82		0.29	18	9.22 (5.2)	2.07	
	CO	19	20.63 (3.0)	18	15.78 (7.0)	0.88						
BAI	TA	18	10.50 (3.6)	18	9.06 (6.2)	0.27	0.33	0.00	17	8.35 (7.4)	0.30	0.02
	ST	20	13.65 (7.8)	18	11.39 (7.9)	0.37		−0.33	18	8.50 (7.5)	0.75	
	CO	19	10.95 (5.9)	18	9.06 (6.3)	0.24						
QOLI	TA	18	−0.47 (1.2)	18	0.41 (1.8)	0.54	0.48	0.63	17	0.98 (2.0)	0.77	−0.30
	ST	20	0.42 (1.7)	18	1.24 (1.7)	0.43		0.11	18	1.51 (1.6)	0.60	
	CO	19	0.83 (1.2)	18	1.42 (1.4)	0.44						

*Abbreviations*: TA: Tailored ICBT; ST: Standardized ICBT; CO: Control group; Pre: Pre-treatment, Post: Post-treatment; FU: 6 month follow-up; BDI–II: Beck Depression Inventory-II; MADRS-S: Montgomery-Åsberg Depression Rating Scale – Self-rated version; BAI: Beck Anxiety Inventory; QOLI: Quality of Life Inventory.

### Measures of depression

Mixed-effects model analyses on the BDI–II and the MADRS-S revealed significant interaction effects of group and time, indicating a difference between groups from pre-treatment to post (*F*(2, 317.6)  = 11.7 and *F*(2, 325.4)  = 11.9 for the BDI–II and the MADRS-S respectively, both *p's*<.001). At post-treatment there were, as seen in Table 2, large effect sizes between tailored treatment and control (post-hoc *t's*>3.46 and *p's*<.001) and moderately large effect sizes between standardized treatment and control (post-hoc *t's*>2.41 and *p's*<.05). Within-group effect sizes were at post-treatment and at follow-up around *d* = 1.5 for the tailored treatment and *d* = 1.0 for the standardized treatment.

The effect sizes between ICBT groups at post and at follow-up were in the range 0.19 to 0.27 and mixed-effects model analyses failed to reveal significant interaction effects of group and time. There was however, a close to significant interaction effect of group and time on the BDI–II (*F*(1,274.5)  = 3.48, *p* = .063) from pre-treatment to follow-up.

Larger differences between ICBT groups were seen in the subgroup with higher initial depression severity. Mixed-effects model analyses revealed significant interaction effects of group and time on the BDI–II (*F*(1, 102.4)  = 6.19, *p*<.05) and on the MADRS-S (*F*(1, 101.2)  = 5.00, *p*<.05) from pre-treatment to post-treatment, favoring the tailored treatment compared to the standardized approach. This is mirrored by between-group effect sizes of *d*  = 0.69 and *d* = 0.82 on the BDI–II and the MADRS-S respectively, as seen in Table 2.

On the contrary, when investigating the lower severity group, no interaction effects of group and time was found between the three groups from pre-treatment to post-treatment or between the ICBT groups from pre-treatment to follow-up. This indicates that the online discussion group was about as effective as the ICBT treatments for the less severely depressed participants.

### Measures of anxiety and quality of life

The results from the BAI and the QOLI are shown in Table 2. Mixed effects model analyses showed significant interaction effect of group and time on both measures from pre-treatment to post-treatment (*F*(2, 285.5)  = 6.93 and *F*(2, 284.5)  = 5.59 for the BAI and the QOLI respectively, both *p's*<.01). No significant interaction effect of group and time could be observed on these measures when comparing the two ICBT treatments, neither in the total sample nor in the subgroup with higher baseline depression scores. Similar to the above results for measures of depression, no interaction effects of group and time were found between the three groups from pre-treatment to post among the less severely depressed participants.

### Recovery rates and clinical global impression

As seen in Table 3, there were 16 out of 36 (44.4%) from the tailored treatment who had recovered from depression at post-treatment. In the standardized treatment and in the control group the proportion of recoverers were 26.5% (11 out of 37) and 7.7% (4 out of 42), respectively. At post-treatment, there was a significant difference between the groups (χ^2^(2, N = 115)  = 12.22; *p*<0.01). Comparing the ICBT groups at post did not reveal a significant difference in recovery. However, among participants with higher baseline scores on depression, there were 9 out of 18 (50.0%) from the tailored treatment who had recovered at post, compared to only 3 out of 17 (17.6%) from the standardized treatment, resulting in a significant difference χ^2^(1, N = 35)  = 4.06; *p*<0.01. At follow-up the differences were no longer significant. Among the less severely depressed participants, no significant differences between groups were found in terms of recovery.

**Table 3:** The proportion of participants reaching recovery and clinical global improvement.

	Tailored		Standardized		Control		χ^2^	
	n	Percent	n	Percent	n	Percent	TA vs ST vs CO	TA vs ST
Total								
Recovered post	16	44.4	11	29.7	4	9.5	12.22**	1.70
Recovered FU	16	44.4	15	40.5				0.11
CGI-I improved post	21	61.8	19	59.4	5	13.9	20.66***	0.04
CGI-I improved FU	25	71.4	22	64.7				0.36
High level of depression								
Recovered post	9	50.0	3	17.6	1	4.3	12.41**	4.06*
Recovered FU	6	33.3	2	11.8				2.31
CGI-I improved post	11	64.7	8	53.3	1	5.3	15.08***	0.43
CGI-I improved FU	11	64.7	9	56.3				0.25
Low level of depression								
Recovered post	7	38.9	8	40.0	3	15.8	3.29	0.005
Recovered FU	10	55.6	13	65.0				0.35
CGI-I improved post	10	55.6	12	63.2	4	23.5	6.24*	0.22
CGI-I improved FU	14	73.7	14	70.0				0.065

*Abbreviations*: TA: Tailored ICBT; ST: Standardized ICBT; CO: Control group; Pre: Pre-treatment, Post: Post-treatment; FU: 6 month follow-up; CGI-I: Clinical Global Impression – Improvement Scale.

Of the 115 participants who were analyzed, 102 were reached for a post-treatment interview and CGI-I measure. At follow-up 104 participants were reached. The proportions of participants who were considered much or very much improved are shown in Table 3.

### Adherence and therapist time

Adherence to the treatment was measured by the amount of finished modules per those prescribed. A module was considered to be finished only if the exercises of the module were completed. The average percentage finished was 77.2% in the tailored group and 80.7% in the standardized group *t*(71)  = 0.54; *p* = .59. As expected, since most participants from the tailored treatment received more modules, the average therapist time per participant was larger in the tailored group (95.2 minutes) compared to the standardized group (74.1 minutes). This difference was significant *t*(71)  = 2.74; *p*<.05. When comparing the number of minutes per prescribed module, the difference between tailored (9.7 minutes/module) and standardized treatment (9.3 minutes/module) was no longer significant *t*(71)  = 0.52; *p* = .60.

## Discussion

The overall aim of this study was to investigate if tailored ICBT is beneficial for a heterogeneous group of people with major depressive disorder, where comorbid symptoms were highly prevalent. The main finding is that the participants who received tailored and standardized ICBT improved more than the participants who were part of a moderated online discussion group with a focus on depression. Despite trends indicating an advantage of tailored ICBT compared to standardized ICBT, no significant differences between treatments were found.

This study also explored how different levels of initial depression severity could moderate response to different treatments. High baseline depression severity was associated with more comorbidity, e.g. more anxiety and worse quality of life. For participants with higher severity, tailored treatment worked better than both the standardized treatment and the online discussion group, both in terms of reduction of depressive symptoms and recovery from depression. In contrast, for participants with lower initial severity, there were no differences in efficacy between the moderated online discussion group and the ICBT treatments.

These results call for an explanation of the active mechanisms in different forms of guided self-help treatment for depression. The exact workings of the different variants of guided self-help are largely unknown, but it seems plausible that the tailored treatment provides a larger set of possible mechanisms. For example, scheduled worry time is a standard component of CBT for generalized anxiety disorder, but is not included in standardized CBT protocols for depression. Hence a patient with a diagnosis of depression and GAD is likely to benefit more from a tailored treatment, which in this case could possibly operate both by e.g. challenging negative core beliefs and by scheduling worrying. The absence of difference in efficacy between conditions for patients of lower severity, could mean that specific mechanisms beyond e.g. expectancy are not active when treating low-severity patients. If this indeed is the case, it would be in line with recent studies on the treatment mechanisms beyond placebo in antidepressants [9].

There are limitations of the study that need to be mentioned. One of the most obvious limitations is that the study was underpowered to detect significant overall differences between the two ICBT treatments, even if trends of between-group effects were found on the BDI–II. A post-hoc power analysis of post-treatment data on the BDI–II between the ICBT treatments revealed that, assuming an α-level of .05, one would need a between-group effect size of *d* = 0.69 to achieve 80% power.

Another limitation is the choice of outcome measures. Even if all outcome measures are established measures of depression, anxiety and quality of life, the BAI has been criticized for measuring primarily symptoms of panic [38]. Thus, BAI does probably not capture all comorbidity in the sample. Finding a balance between a few global or many specific measures is a challenge for future research on tailored and unified treatments targeting more than one specific disorder.

A related limitation is the measure of comorbidity. In the present study, a set of online screening questions with a decision tree structure were used to detect comorbid problems. While preserving the structure from the PrimeMD, it is still unknown if this data is a valid measure of comorbidity. Future research should investigate the validity of such a screening system and how accurate it can be in the process of identifying psychiatric diagnoses.

A further limitation concerns the therapists in the study who all were psychologists in training, albeit during the last semester of training in a five year program. There is some evidence that students are less effective as therapists when conducting face-to-face therapy [39]. Therefore, it is possible that experienced therapists would have performed even better. This hypothesis can be contrasted with recent indications that a computer technician can conduct ICBT as good as a clinician [40], [41]. These recent results call for further research on who can conduct ICBT.

Some clinical implications of this study are discussed as follows. Since comorbidity is so common with major depression, one important implication is that tailoring may be a way of addressing comorbidity while conducting ICBT for major depression, at least for more depressed patients. While tailoring may add a small treatment effect, it is still reasonable that standardized treatments or maybe even online discussion groups are the best options to use when the client's problems are captured by a single diagnosis or when the problems are subclinical. Future research should investigate under which conditions tailored treatment is preferable to standardized treatment.

From a cost-effectiveness perspective, there are probably differences among the treatments tested in this trial. Tailoring, as it is conducted in this study, involves prescribing a set of modules after a thorough assessment. This procedure naturally takes more time from the clinician than just assigning a fixed treatment package which is independent of symptom profile. However, this procedure may be automatized and there are also indications that patients can prescribe a similarly effective treatment as a clinician [42]. Further, from a cost-effectiveness perspective, the results from this study indicate that tailored treatment with a focus on comorbidity may be a way to reach out to patients with more severe forms of depression. Depression with higher severity is associated with even more disability for the patient and also larger costs for society [43]. Future ICBT research should focus on conducting larger trials on treatments for this group of patients.

In summary, this study is one of the first to test a tailored approach to ICBT for depression and the first to test it directly compared to a standardized treatment package. The tailored treatment in the present study targeted comorbidity, which is commonly associated with major depression. Results from the study indicate that ICBT that addresses comorbidity by tailoring may be more effective than a standardized approach for patients with higher depression severity at baseline. Future studies should focus on when to choose tailored treatment instead of standardized treatment and on dissemination issues, for example how tailored ICBT works in real life settings, e.g. in psychiatry and primary care.

## Supporting Information

Checklist S1 **CONSORT checklist.**
(DOC)

Protocol S1 **Trial Protocol.**
(DOC)
