# Supplementary material for: Tailored vs. Standardized Internet-Based Cognitive Behavior Therapy for Depression and Comorbid Symptoms: A Randomized Controlled Trial
Source: PLoS One. 2012 May 15;7(5):e36905. doi: 10.1371/journal.pone.0036905 (PMC3352859; doi:10.1371/journal.pone.0036905)
Supplement: Protocol S1 — Trial Protocol. (DOC) [file pone.0036905.s002.doc]

**Research plan**

**Background**

According to the National Council on Technology Assessment (2004) the economic costs of depression are high. A calculation performed in 1997 showed that the direct and indirect costs of depression amounted to 12 billion in one year. Depression is very common compared to other medical diagnoses - an estimation is that 15-17% are affected at some time during their lifetime (Ebmeier, 2006).A large number of people with depression are not detected and those detected, are often given the wrong treatment (Kessler, 2003).

Research has shown that co-morbid conditions are very common in depression. For example, over 50% of people with depression diagnosis also meet diagnostic criteria for anxiety disorder and social phobia (Kessler et al., 2007).Comorbid somatic condition also occurs (Pincus & Williams, 1999).

Both pharmacological and psychotherapeutic treatment has been shown to have proven efficacy in treating depression (SBU, 2004). There is some evidence that psychological treatment results in fewer relapses in depression than medical treatment after 18 months of treatment (SBU, 2004). Internet-based CBT treatment of depression has been shown effective in previous studies (Andersson et al., 2005).

*Why Internet therapy?*

Therapy over the Internet is a so-called distance therapy, meaning that the client and the therapist does not meet physically (Skinner & Zack, 2004). Internet therapy occurred in 1979 in conjunction with a digital network launched in the U.S., then in the form of various self-help and support groups. In 1995, a number of therapy clinics became available through the Internet. According to Skinner and Zack (2004), therapy over the internet is an effective and ethical psychological treatment.

The combination of CBT and self-help therapy via the Internet has been successfully tested in several studies where the aim has been to treat and relieve mental disorders, including depression, social phobia and panic disorder (Andersson et al., 2005, 2006; Carlbring et al., 2001 for a review, see Andersson, Carlbring et al. 2006

# Purpose and potential benefits of the study

The purpose of the current Internet treatment is to conduct a randomized controlled treatment study targeted to people with depression and various comorbid conditions, such as anxiety disorders. The study is based on the same proven CBT techniques as in previously implemented Internet services. In earlier Internet-based CBT treatment for depression, individuals were excluded if there have been co-morbid with other conditions. The present study aims to develop and implement a customized treatment plan according to each person's individual needs and problems.

One of the reasons for conducting this study is to investigate whether treatment via the Internet can help people suffering from depression and thereby pose an accessible, cost effective and flexible complement to traditional treatment. The motive is to deepen knowledge about the treatment through the Internet, and to acquire knowledge about the Internet Therapy for anxiety problems. The results can provide information about whether CBT treatment through the Internet may be another treatment option for depression. If treatment should prove to be effective, this would mean an addition to traditional psychotherapy and psychopharmacological treatment currently available for patients with this problem.

**Patient Selection and implementation**

The study will be advertised on the internet and through newspapers. After reading the presentation on our website, the subject notify their interest and complete the screening form on the website that is sent to study leader. Subjects were also writes under a consent under the Personal Data Act (PUL) and sends it to the researchers. For screening, we intend to make use of the Beck Depression Inventory-II (BDI-II), Montgomery Åsberg Depression Rating Scale - short version (MADRS-S) and Beck Anxiety Inventory (BAI).As for MADRS-S, we will exclude those with high scores on the question regarding suicidal ideation (question 9). For the other measures, there are no upper or lower limit. However, only people over 18 and those who do not attend a concurrent psychological therapy are included. People who meet the inclusion criteria from the screening via the Internet will be contacted by phone to confirm any psychiatric diagnosis with a clinically structured interview (SCID). Treatment history and prior treatments will also be covered.This also provides an opportunity to determine what treatment the participant will get. A licensed physician (specialist in psychiatry) will review the results from the screening and from the structured interview. After this the participant will be notified of inclusion or exclusion. This procedure allows us to ensure that people with less suitable problems (eg psychological reactions to medications) are not included. Persons with no unequivocal psychiatric diagnosis (e.g. mixed subclinical symptoms) may be included.Addition to the above measures, we will make use of Quality of Life Inventory (QOLI) before and after treatment to measure the life quality.

After inclusion, participants will be randomized to one of the three groups. The first active treatment offer a modular program with 10 treatment modules which to some extent may vary with the exception of the first and the last module. Decisions on which modules will be made by a licensed psychotherapist and project leader with the interview as a basis and in consultation with the student who conducted the interview. In the second active treatment group given a previously tested CBT treatment via the Internet (Andersson et al., 2005) where treatment follows a predetermined structure and the modules are given in a previously determined order.

If necessary, the project's psychiatrist is consulted. Processing modules are retrieved from previous projects targeting social anxiety disorder, panic disorder, generalized anxiety and depression. The modules are akin to therapy sessions and homework assignments will be provided adjacent to each module. Each participant receives a therapist who is supervised by a psychotherapist / supervisor and physician. The method is well proven in more than 20 studies in Sweden and internationally.  When the end of therapy for the treatment groups is reached, the control group will have the opportunity to take part of the self-help program. The control group will not be left alone during the waiting period but offered to participate in a virtual discussion group run by the project (ie, closed groups that require passwords). This procedure has been included in previous studies in Uppsala and has been appreciated by the participants.

#

# Time schedule and Evaluation

Preparations will be made during spring 2009. This means the revision of manuals for self-help programs, and training of therapists. A website will also be created and then used during the study. People who have registered for the study and screened will be interviewed, later in the period August 2009. After randomization to experimental groups and control group, treatment begins which is expected to last for ten weeks. After stopping treatment, the data will be processed and compiled.

**Project team**

Gerhard Andersson, Professor, lic. psychologist, IBL Linköping University

Per Carlbring, associate professor, lic. psychologist, IBL, Linköping University

Andréas Rousseau, MD., Specialist in psychiatry. Psychiatric clinic, University Hospital of Linköping.

Robert Johansson, psychologist candidate, IBL Linköping University

Erik Johnsson, psychologist candidate, IBL Linköping University

Elin Sjöberg, psychology candidate, IBL Linköping University

Magnus Sjögren, psychology candidate, IBL Linköping University

**References**

Andersson, G., Bergström, J., Holländare, F., Carlbring, P., Kaldo, V., & Ekselius, L.

(2005). Internet-based self-help for depression: a randomised controlled trial.

*British Journal of Psychiatry, 187*, 456-461.

Andersson, G., Carlbring, P., Holmström, A., Sparthan, E., Furmark, T., Nilsson-

Ihrfelt, E., Buhrman, M., & Ekselius, L. (2006). Internet-based self-help with

therapist feedback and in-vivo group exposure for social phobia: a randomized

controlled trial. *Journal of Consulting and Clinical Psychology, 74*, 677-686.

Andersson, G., Carlbring, P., & Kaldo, V. (2006). Kognitiv beteendeterapi via

internet - en behandlingsform för framtiden? *Psykisk Hälsa* (1), 50-58.

Carlbring, P., Westling, B. E., Ljungstrand, P., Ekselius, L., & Andersson, G. (2001).

Treatment of panic disorder via the Internet- a randomized trial of a self-help

program. *Behavior Therapy, 32*, 751-764.

Ebmeier, K. P., Donaghey C., & Steele J. D. (2006). Recent developments and current controversies in depression. *Lancet, 367,* 153-167*.*

Kessler, R. C., Berglund P., Demler O., Jin, R., Koretz, D., Merikangas, K. R., Rush, A. J., Walters, E. E., & Wang, P. S. (2003). The epidemiology of major depressive disorder: results from the National Comorbidity Survey Replication (NCS-R). *Journal of the American Medical Association, 289,* 3095-3105

Kessler, R. C., Merikangas, K. R., & Wang, P. S. (2007). Prevalence, comorbidity, and service utilization for mood disorders in the United States at the beginning of the twenty-first century. *Annual review of clinical psychology*, *3*, 137-158.

Pincus, T., & Williams, A. (1999). Models and measurements of depression in chronic pain. *Journal of Psychosomatic Research*, *47*, 211-219.

Skinner, A., & Zack, J. (2004). Counseling and the internet. *American behavioral*

*scientist, 48(4),* 434-446*.*

Statens beredning för medicinsk utredning. (2004). *Behandling av depressions-sjukdomar – En systematisk litteraturöversikt. Sammanfattning och slutsatser*. Stockholm: SBU*.*
